# Development and validation of a preoperative nomogram to predict lymph node metastasis in patients with bladder urothelial carcinoma

**DOI:** 10.1007/s00432-023-04978-7

**Published:** 2023-06-15

**Authors:** Junjie Ji, Yu Yao, Lijiang Sun, Qingya Yang, Guiming Zhang

**Affiliations:** 1 Department of Urology, The Affiliated Hospital of Qingdao University, Qingdao, China; 2 Department of Urology, Qilu Hospital (Qingdao), Cheeloo College of Medicine, Shandong University, Qingdao, China

**Keywords:** Bladder cancer, Lymph node metastasis, Bladder urothelial carcinoma, Cancer risk, Nomogram, External validation

## Abstract

**Purpose:**

Predicting lymph node metastasis (LNM) in patients with bladder urothelial carcinoma (BUC) before radical cystectomy aids clinical decision making. Here, we aimed to develop and validate a nomogram to preoperatively predict LNM in BUC patients.

**Methods:**

Patients with histologically confirmed BUC, who underwent radical cystectomy and bilateral lymphadenectomy, were retrospectively recruited from two institutions. Patients from one institution were enrolled in the primary cohort, while those from the other were enrolled in the external validation cohort. Patient demographic, pathological (using transurethral resection of the bladder tumor specimens), imaging, and laboratory data were recorded. Univariate and multivariate logistic regression analyses were performed to explore the independent preoperative risk factors and develop the nomogram. Internal and external validation was conducted to assess nomogram performance.

**Results:**

522 and 215 BUC patients were enrolled in the primary and external validation cohorts, respectively. We identified tumor grade, infiltration, extravesical invasion, LNM on imaging, tumor size, and serum creatinine levels as independent preoperative risk factors, which were subsequently used to develop the nomogram. The nomogram showed a good predictive accuracy, with area under the receiver operator characteristic curve values of 0.817 and 0.825 for the primary and external validation cohorts, respectively. The corrected C-indexes, calibration curves (after 1000 bootstrap resampling), decision curve analysis results, and clinical impact curves demonstrated that the nomogram performed well in both cohorts and was highly clinically applicable.

**Conclusion:**

We developed a nomogram to preoperatively predict LNM in BUC, which was highly accurate, reliable, and clinically applicable.

## Introduction

According to a 2020 report, bladder cancer (BC) is the tenth most common malignancy overall and the sixth most common malignancy in men worldwide (Sung et al. 2021). Bladder urothelial carcinoma (BUC) constitutes over 90% of BC cases (Hsieh et al. 2023). Moreover, ~ 75% of BC patients, in whom BC is confined to the mucosa or submucosa, are defined as having non-muscle‑invasive bladder cancer (NMIBC) (2017); these patients are typically treated with transurethral resection of the bladder tumor (TURBT), followed by intravesical chemotherapy or Bacillus Calmette-Guérin vaccine administration (Babjuk et al. 2022). However, even after these treatments, recurrence or progression occur in nearly 40% of high-risk or very high-risk patients (Kamat et al. 2018). Although muscle-invasive bladder cancer (MIBC) accounts for only a small proportion of BC cases, it is associated with a higher risk of cancer-specific mortality in relation to NMIBC (Burger et al. 2013).

Radical cystectomy (RC) plus pelvic lymph node (LN) dissection (LND) and urinary diversion are the standard treatment options for MIBC patients and some patients with high-grade or very high-grade NMIBC (Lenis et al. 2020a, b). However, the development of lymph node metastasis (LNM) in BUC patients is one of the most valuable indicators of poor prognosis and tumor invasiveness (May et al. 2011; Shariat et al. 2012). The 5-year overall survival rate of BUC patients with LNM following RC treatment alone is only 19%. The combination of RC with neoadjuvant or adjuvant chemotherapy raises the 5-year overall survival rate to 31% and 26%, respectively, which is still inadequately low (Galsky et al. 2016). It was reported that nearly 8% of NMIBC patients and 25% of MIBC patients develop LNM (Lenis et al. 2020a, b). Nevertheless, BUC patients with LNM can still achieve prolonged survival with appropriate treatment measures before they develop distant metastases (Darwish et al. 2020). LND typically involves the removal of nodal tissue up to the common iliac bifurcation. Although one prospective randomized trial in BC patients found no significant survival advantage in extended LND, a trend towards a survival benefit was observed (Gschwend et al. 2019). Several reviews also suggested that extended LND prolonged survival in a subset of BUC patients with LNM (Bruins et al. 2014, Ghodoussipour and Daneshmand 2019). However, the complication rates of RC, classed according to the Clavien-Dindo Classification system, ranged from 50 to 87.5% (for grades 1–4) and the severe complication rate ranged from 30 to 42% (for > grade 3) (Cicione et al. 2020; Demaegd et al. 2020; Furrer et al. 2019; Haas et al. 2021). Some selected MIBC patients were reported to gain quality-adjusted life years after receiving bladder-sparing tri-modality therapy (Royce et al. 2019). Besides, RC may be viewed as an overtreatment for some high-grade NMIBC patients with a low risk of progression and metastasis (Klaassen et al. 2018). Therefore, it is vital to preoperatively predict LNM in clinical decision.

Several studies have extensively researched the role of nomograms in predicting LNM in BC to aid clinical decision making. While some of these studies used radiomic (Wu et al. 2017, 2018a, b) or genomic data (Lu et al. 2019, Wu et al. 2018a, b) to develop a preoperative nomogram, the difficulty of collecting suitable information restricts the clinical use of such a nomogram. Meanwhile, two other nomograms were designed to predict non-organ-confined BUC prognosis by including clinical and pathological information from BUC patients; however, their accuracy was limited by a selection bias introduced as a result of a small sample size and the use of data from a single institution (Green et al. 2013; Xie et al. 2012). A large-scale multicenter nomogram was constructed using demographic and pathological data from the Surveillance, Epidemiology, and End Results (SEER) database (Tian et al. 2021). However, the area under the receiver operating characteristic (ROC) curve (AUC) for this nomogram in the training cohort was only 0.69, indicating its low accuracy at predicting the development of LNM in BC patients after RC. Hence, in the present study, we aimed to develop and validate a preoperative nomogram for predicting LNM in BUC using patient demographic information, pathologic characteristics from TURBT specimens, imaging data, and laboratory measurements.

## Materials and methods

### Patients and patient characteristics

This retrospective study included patients with histologically confirmed BUC, who underwent RC and bilateral lymphadenectomy at the Affiliated Hospital of Qingdao University (between January 2016 and April 2022) and Qingdao Campus of Qilu Hospital of Shandong University (between January 2014 and December 2022). The exclusion criteria were as follows: (a) age < 18 years; (b) patients who did not undergo TURBT before RC or those without muscle tissue in their TURBT specimen; (c) patients with distant metastasis; (d) patients with incomplete imaging examination data before RC; (e) patients with tumors that originated at sites other than the bladder; (f) patients with incomplete laboratory measurements, collected within 1 month before RC; (g) patients with severe inflammation or immune system diseases; (h) patients receiving preoperative radiotherapy; (i) patients with severe or end-stage chronic kidney disease; and (j) patients with RC specimens which were not pathologically confirmed as BUC.

The following patient characteristics were recorded: (a) demographics, including age, sex, and body mass index; (b) pathologic TURBT characteristics before RC, including tumor grade, papillary tumor presence, urothelial variants, muscle invasion, and infiltration; (c) imaging characteristics, including hydronephrosis, extravesical invasion, LNM on imaging, and tumor size; and (d) laboratory measurements, including neutrophil count, monocyte count, basophil count, eosinophil count, lymphocyte count, erythrocyte count, platelet count, hemoglobin, fibrinogen, urea nitrogen, creatinine, and albumin. If patients underwent several rounds of TURBT, the pathological characteristics of the highest tumor grade or cancer stage were recorded.

The neutrophil to lymphocyte ratio (NLR), platelet to lymphocyte ratio (PLR), monocyte to lymphocyte ratio (MLR), and neutrophil to platelet ratio (NPR) were calculated from the respective cell counts. The systemic immune-inflammation index (SII) was calculated by multiplying the platelet count by the neutrophil count and dividing this value by the lymphocyte count. Pre-RC laboratory measurements were collected in cases when the latest TURBT was performed over 1 month before RC; otherwise, these measurements were collected before TURBT to reduce the impact of surgery on the results.

### Independent risk factors for LNM and nomogram construction

Patients from the Affiliated Hospital of Qingdao University were assigned to the primary cohort, while those from Qingdao Campus of Qilu Hospital of Shandong University were assigned to the external validation cohort. Univariate and multivariate logistic regression analyses were performed to explore the independent preoperative risk factors for LNM in BUC. The significant risk factors identified in the univariate analysis of the primary cohort data were included in the multivariate logistic regression model. The nomogram of LNM in BUC was then established based on the significant risk factors identified in multivariate logistic regression model.

### Internal and external validation of the nomogram

Next, the nomogram-associated ROC curve was plotted, and AUC was determined. Discrimination was assessed using Harrell’s C-index. Bootstrap resampling validation (with 1000 bootstrap resamples) was used to calculate a corrected C-index. The Hosmer–Lemeshow goodness-of-fit test and calibration curve were used to assess the calibration of the nomogram. Decision curve analysis (DCA) was performed to demonstrate net benefit for each risk threshold probability, as well as the clinical application value of the nomogram. In addition, the clinical impact curve was plotted to demonstrate the potential benefit of using the nomogram in clinical practice.

External validation was performed by applying the established internal nomogram model to the validation cohort. We therefore compared the ROC curves, AUC, Harrell’s C-indexes, corrected C-indexes, calibration curves, DCA results, and the clinical impact curves of the primary and validation cohorts to verify the stability of the nomogram.

### Statistical analyses

Frequencies and proportions were used to describe categorical variables, means and standard deviations were used for continuous variables with a normal distribution, and medians and interquartile ranges were used for continuous variables with an abnormal distribution. Student's t test, Mann–Whitney U test, and Chi-squared test were used in the univariate analysis of continuous variables with a normal distribution, continuous variables with an abnormal distribution, and categorical variables, respectively. Forward step-wise selection was applied in the multivariate logistic regression analysis. The nomogram, ROC curve, calibration curve, DCA, and clinical impact curve were constructed/performed using the R software packages rms, pROC, and rmda. Statistical analyses were performed using SPSS (version 24.0) and R software (version 4.1.0). A bilateral *P* value < 0.05 was considered as a measure of statistical significance.

## Results

### Patient selection and characterization

**Fig. 1:**
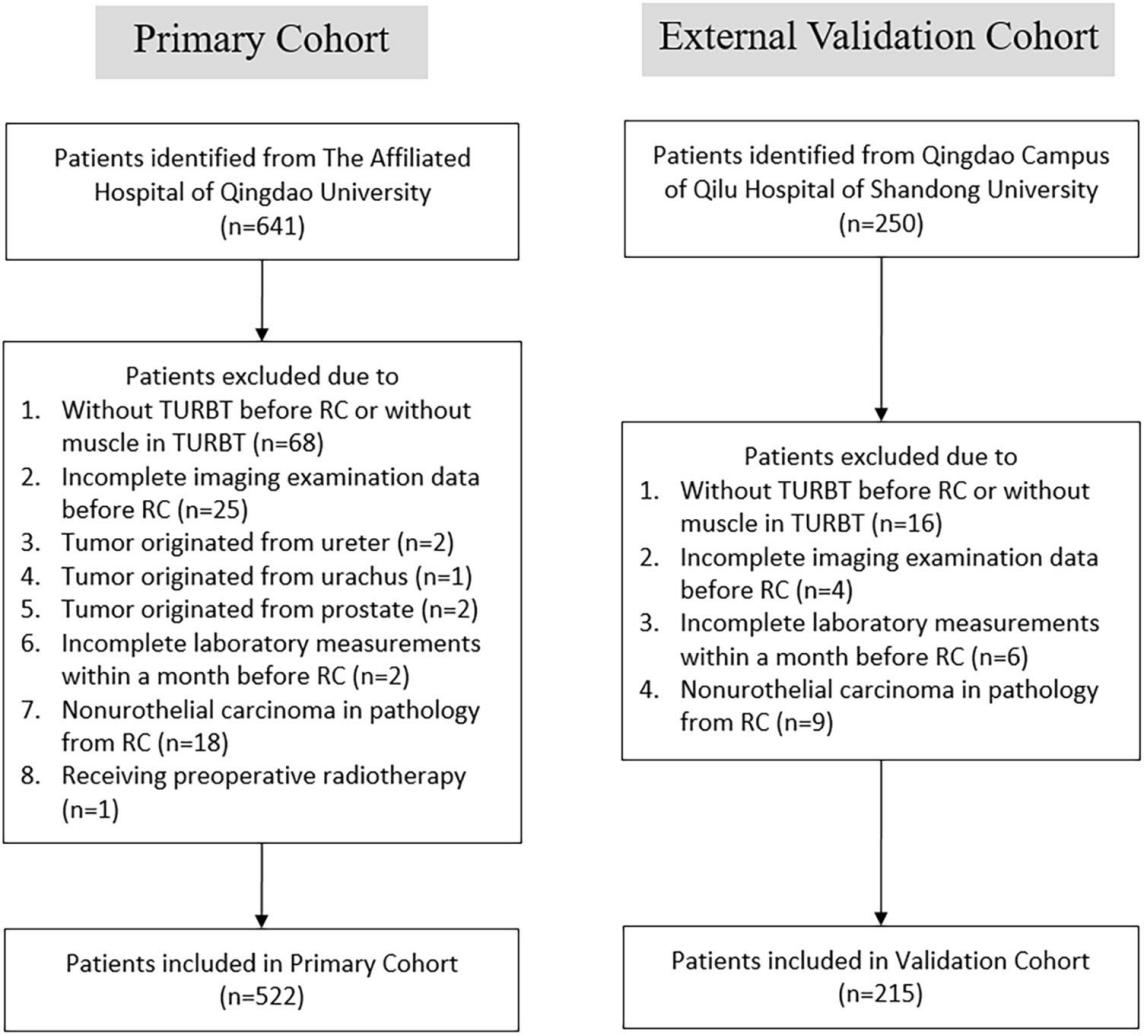
Flowchart showing the patient selection process for the primary and external validation cohorts. *TURBT* transurethral resection of the bladder tumor, *RC* radical cystectomy

A total of 641 potentially eligible BUC patients were identified from the Affiliated Hospital of Qingdao University cohort, while 250 patients were identified from the Qingdao Campus of Qilu Hospital of Shandong University cohort. After selection, we enrolled 522 BUC patients (85 patients with LNM and 437 patients without) in the primary cohort and 215 BUC patients (35 patients with LNM and 180 patients without) in the validation cohort (Fig. 1).

Patient characteristics and the results from the univariate analyses of both cohorts are presented in Table 1. The rate of LNM was not significantly different between the primary and validation cohorts (χ2 test, P = 0.999). The univariate analysis of data from BUC patients with or without LNM in the primary cohort showed that those with LNM were significantly older (P = 0.017) and had the following characteristics: higher grade tumors (P < 0.001), which were not of papillary nature (P < 0.001); more extensive tumor infiltration (P < 0.001); more possibility of hydronephrosis (P < 0.001) and extravesical invasion (P < 0.001); more evidence of LNM on imaging (P < 0.001); larger tumors (≥ 4 cm) (P < 0.001); higher neutrophil count (P = 0.007); lower erythrocyte count (P = 0.027); higher platelet count (P = 0.034); lower hemoglobin (P = 0.011); higher fibrinogen (P < 0.001); higher serum creatinine (P = 0.004); lower albumin (P = 0.012); higher NLR (P = 0.001); higher PLR (P = 0.021); higher MLR (P = 0.003); and higher SII (P = 0.001).

**Table 1:** Baseline characteristics of the cohort by lymph node status

Characteristics	Primary cohort (*n* = 522)			Validation cohort (*n* = 215)		
	LNM (–)	LNM (+)	*P*	LNM (–)	LNM (+)	*P*
	437	85		180	35	
Demography						
Age	65.19 ± 8.80	67.68 ± 8.76	0.017^*^	65.64 ± 10.03	66.60 ± 8.25	0.595
Sex			0.369			0.224
Male	369 (84.4%)	75 (88.2%)		150 (83.3%)	32 (91.4%)	
Female	68 (15.6%)	10 (11.8%)		30 (16.7%)	3 (8.6%)	
BMI	24.05 ± 2.99	23.35 ± 3.38	0.076	24.43 ± 3.05	24.37 ± 3.48	0.935
Pathology						
Grade			< 0.001^***^			0.002^**^
High grade	335 (76.7%)	83 (97.6%)		130 (72.2%)	34 (97.1%)	
Low grade	102 (23.3%)	2 (2.4%)		50 (27.8%)	1 (2.9%)	
Papillary			< 0.001^***^			0.007^**^
Yes	183 (41.9%)	12 (14.1%)		80 (44.4%)	7 (20.0%)	
No	254 (58.1%)	73 (85.9%)		100 (55.6%)	28 (80.0%)	
Urothelial variants			0.56			0.199
Yes	33 (7.6%)	8 (9.4%)		19 (10.6%)	7 (20.0%)	
No	404 (92.4%)	77 (90.6%)		161 (89.4%)	28 (80.0%)	
Muscle Invasion			0.331			< 0.001^***^
Yes	37 (8.5%)	10 (11.8%)		15 (8.3%)	17 (48.6%)	
No	400 (91.5%)	75 (88.2%)		165 (91.7%)	18 (51.4%)	
Infiltration			< 0.001^***^			0.001^**^
Yes	231 (52.9%)	66 (77.6%)		100 (55.6%)	30 (85.7%)	
No	206 (47.1%)	19 (22.4%)		80 (44.4%)	5 (14.3%)	
Imaging						
Hydronephrosis			< 0.001^***^			< 0.001^***^
Yes	93 (21.3%)	43 (50.6%)		33 (18.3%)	22 (62.9%)	
No	344 (78.7%)	42 (49.4%)		147 (81.7%)	13 (37.1%)	
Extravesical Invasion			< 0.001^***^			< 0.001^***^
Yes	69 (15.8%)	43 (50.6%)		35 (19.4%)	20 (57.1%)	
No	368 (84.2%)	42 (49.4%)		145 (80.6%)	15 (42.9%)	
LNM on Imaging			< 0.001^***^			< 0.001^***^
Yes	41 (9.4%)	31 (36.5%)		9 (5.0%)	9 (25.7%)	
No	396 (90.6%)	54 (63.5%)		171 (95.0%)	26 (74.3%)	
Tumor Size (cm)			< 0.001^***^			< 0.001^***^
≥ 4	146 (33.4%)	52 (61.2%)		58 (32.2%)	24 (68.6%)	
< 4	291 (66.6%)	33 (38.8%)		122 (67.8%)	11 (31.4%)	
Laboratory						
Neutrophil count	3.85 (3.10–5.07)	4.44 (3.38–6.25)	0.007^**^	3.84 (2.86–4.74)	4.39 (3.22–5.63)	0.047^*^
Monocyte count	0.48 (0.37–0.62)	0.53 (0.40–0.65)	0.088	0.49 (0.41–0.60)	0.63 (0.46–0.77)	0.001^**^
Basophil count	0.03 (0.02–0.04)	0.03 (0.02–0.05)	0.663	0.03 (0.02–0.04)	0.04 (0.02–0.05)	0.568
Eosinophil count	0.11 (0.06–0.18)	0.13 (0.07–0.25)	0.051	0.12 (0.07–0.20)	0.18 (0.08–0.25)	0.062
Lymphocyte count	1.80 (1.41–2.25)	1.82 (1.34–2.17)	0.32	1.90 (1.50–2.23)	1.85 (1.55–2.36)	0.779
Erythrocyte count	4.50 (4.16–4.82)	4.33 (3.88–4.70)	0.027^*^	4.52 (4.24–4.96)	4.40 (3.97–4.72)	0.086
Platelet count	225 (187–263)	239 (197–297)	0.034^*^	226 (191–257)	252 (191–268)	0.238
Hemoglobin	139 (127–149)	136 (116–146)	0.011^*^	142 (130–152)	136 (118–146)	0.062
Fibrinogen	3.01 (2.59–3.61)	3.54 (3.04–3.92)	< 0.001^***^	3.00 (2.56–3.43)	3.56 (2.98–4.02)	< 0.001^***^
Urea nitrogen	6.33 (5.11–7.70)	6.30 (5.34–8.16)	0.509	6.17 (5.20–7.53)	6.30 (4.86–7.68)	0.708
Creatinine	78.7	87.1	0.004^**^	82.5	89.7	0.040^*^
	(64.99–92.85)	(68.50–108.10)		(70.00–97.75)	(73.00–108.10)	
Albumin	40.83 ± 4.28	39.53 ± 4.55	0.012^*^	40.66 ± 4.47	39.10 ± 3.58	0.053
NLR	2.11 (1.57–3.04)	2.58 (1.96–3.69)	0.001^**^	1.96 (1.50–2.74)	2.37 (1.67–2.94)	0.12
PLR	126.47	140.49	0.021^*^	121.97	121.08	0.979
	(97.59–158.00)	(110.93–181.01)		(94.72–154.10)	(91.82–163.98)	
MLR	0.27 (0.20–0.36)	0.29 (0.24–0.39)	0.003^**^	0.27 (0.21–0.33)	0.33 (0.24–0.45)	0.020^*^
NPR	0.018	0.019	0.152	0.017	0.019	0.122
	(0.014–0.023)	(0.015–0.025)		(0.013–0.021)	(0.014–0.027)	
SII	475.68	639.44	0.001^**^	455.56	473.81	0.177
	(342.62–698.23)	(417.00–905.00)		(309.68–671.76)	(376.79–814.44)	

*LNM* lymph node metastasis, *BMI* body mass index, *NLR* neutrophil-to-lymphocyte ratio, *PLR* platelet-to-lymphocyte ratio, *MLR* monocyte-to-lymphocyte ratio, *NPR* neutrophil-to-platelet ratio, *SII* systemic immune inflammation index

*P < 0.05

**P < 0.01

***P< 0.001

### Independent risk factors for LNM and nomogram construction

**Fig. 2:**
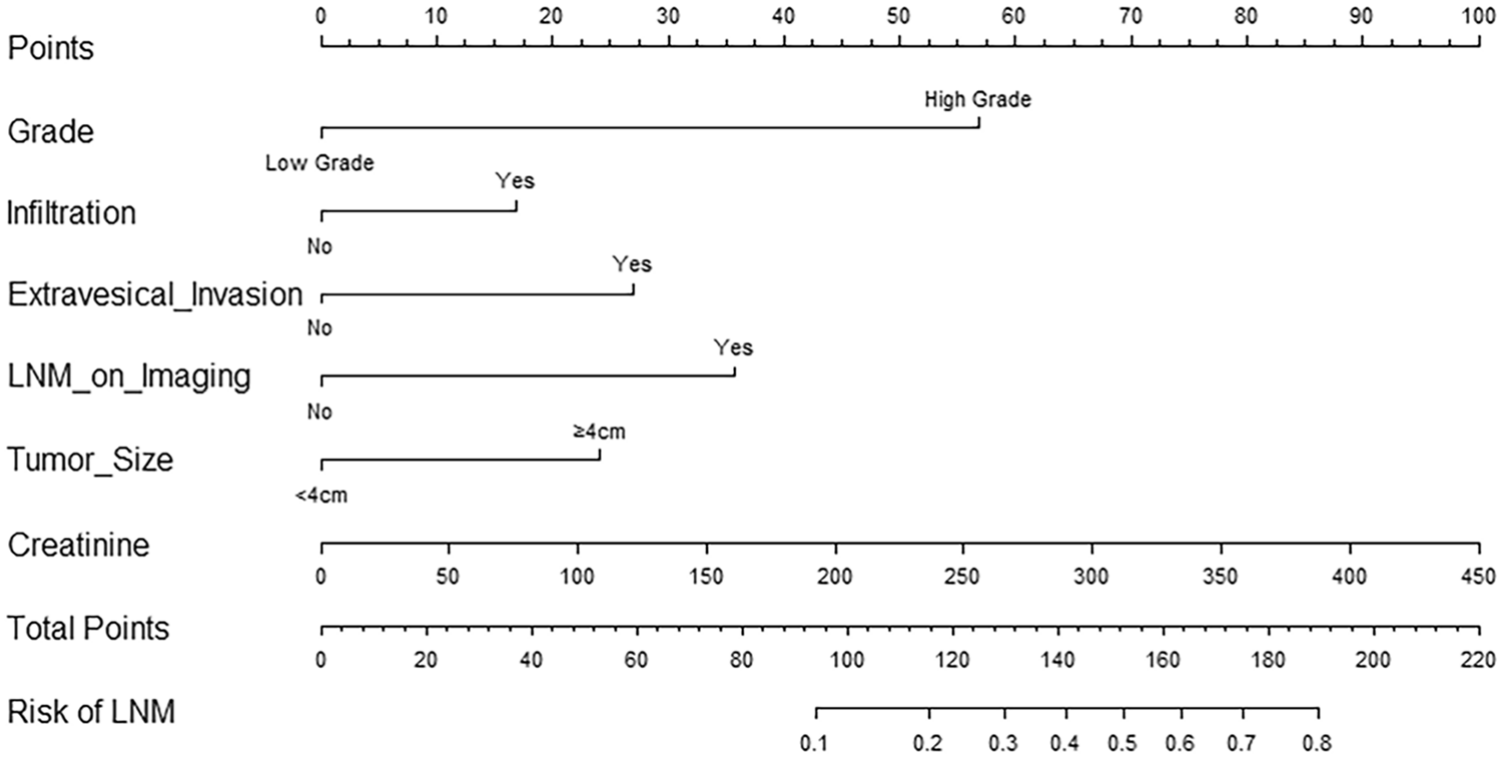
A nomogram for preoperatively predicting LNM in BUC patients, according to tumor grade, tumor infiltration, extravesical invasion, LNM on imaging, tumor size, and creatinine levels. *LNM* lymph node metastasis, *BUC* bladder urothelial carcinoma

Parameters that were identified as significantly different between BUC patients with LNM and those without in the univariate analysis were next included in the binary multivariate logistic regression analysis. We found that high tumor grade (odds ratio [OR] = 8.400, 95% confidence interval [CI] 1.814–38.904, P = 0.007), infiltration (OR = 1.878, 95%CI: 1.001–3.522, P = 0.050), extravesical invasion (OR = 2.743, 95% CI 1.548–4.861, P = 0.001), presence of LNM on imaging (OR = 3.823, 95% CI 2.065–7.078, P < 0.001), larger tumor size (≥ 4 cm) (OR = 2.469, 95%CI: 1.427–4.272, P = 0.001), and higher serum creatinine levels (OR = 1.008, 95% CI 1.000–1.017, P = 0.043) were independent preoperative risk factors for LNM in BUC (Table 2). We pooled these independent risk factors to establish the prediction model and presented the model as a nomogram (Fig. 2).

**Table 2:** Logistic regression analysis of the risk factors for lymph nodes metastasis (Forward LR)

Inflammation indicators	Univariate analysis		Multivariate analysis	
	OR (95% CI)	*P* value	OR (95% CI)	*P* value
Age	1.033 (1.006–1.062)	0.018^*^		
Grade				
High grade	12.636 (3.055–52.271)	< 0.001^***^	8.400 (1.814–38.904)	0.007^**^
Low grade	Reference		Reference	
Papillary				
Yes	0.228 (0.120–0.432)	< 0.001^***^		
No	Reference			
Infiltration				
Yes	3.098 (1.798–5.336)	< 0.001^***^	1.878 (1.001–3.522)	0.050^*^
No	Reference		Reference	
Hydronephrosis				
Yes	3.787 (2.336–6.138)	< 0.001^***^		
No	Reference			
Extravesical invasion				
Yes	5.460 (3.322–8.975)	< 0.001^***^	2.743 (1.548–4.861)	0.001^**^
No	Reference		Reference	
LNM on imaging				
Yes	5.545 (3.211–9.575)	< 0.001^***^	3.823 (2.065–7.078)	< 0.001^***^
No	Reference		Reference	
Tumor size (cm)				
≥ 4	3.141 (1.945–5.072)	< 0.001^***^	2.469 (1.427–4.272)	0.001^**^
< 4	Reference		Reference	
Neutrophil count	1.116 (1.012–1.232)	0.029^*^		
Erythrocyte count	0.621 (0.430–0.899)	0.011^*^		
Platelet count	1.003 (1.000–1.006)	0.092		
Hemoglobin	0.984 (0.974–0.995)	0.004^**^		
Fibrinogen	1.942 (1.469–2.567)	< 0.001^***^		
Creatinine	1.012 (1.005–1.020)	0.001^**^	1.008 (1.000–1.017)	0.043^*^
Albumin	0.934 (0.886–0.986)	0.013^*^		
NLR	1.015 (0.966–1.068)	0.549		
PLR	1.002 (1.000–1.005)	0.074		
MLR	1.381 (0.656–2.910)	0.395		
SII	1.000 (1.000–1.000)	0.220		

*OR* odds ratio, *CI* confidence interval, *LN* lymph node, *NLR* neutrophil-to-lymphocyte ratio, *PLR* platelet-to-lymphocyte ratio, *MLR* monocyte-to-lymphocyte ratio, *SII* systemic immune inflammation index

*P< 0.05

**P < 0.01

***P < 0.001

### Internal validation of the nomogram

**Fig. 3:**
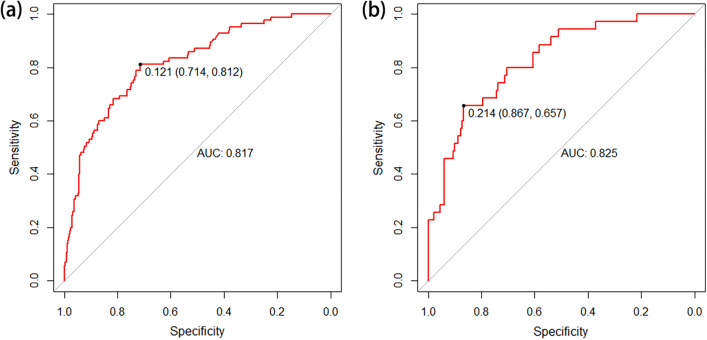
The ROC curves for preoperatively predicting LNM in patients with BUC in the primary (**a**) and external validation (**b**) cohorts. *ROC* receiver operating characteristic, *LNM* lymph node metastasis, *BUC* bladder urothelial carcinoma

**Fig. 4:**
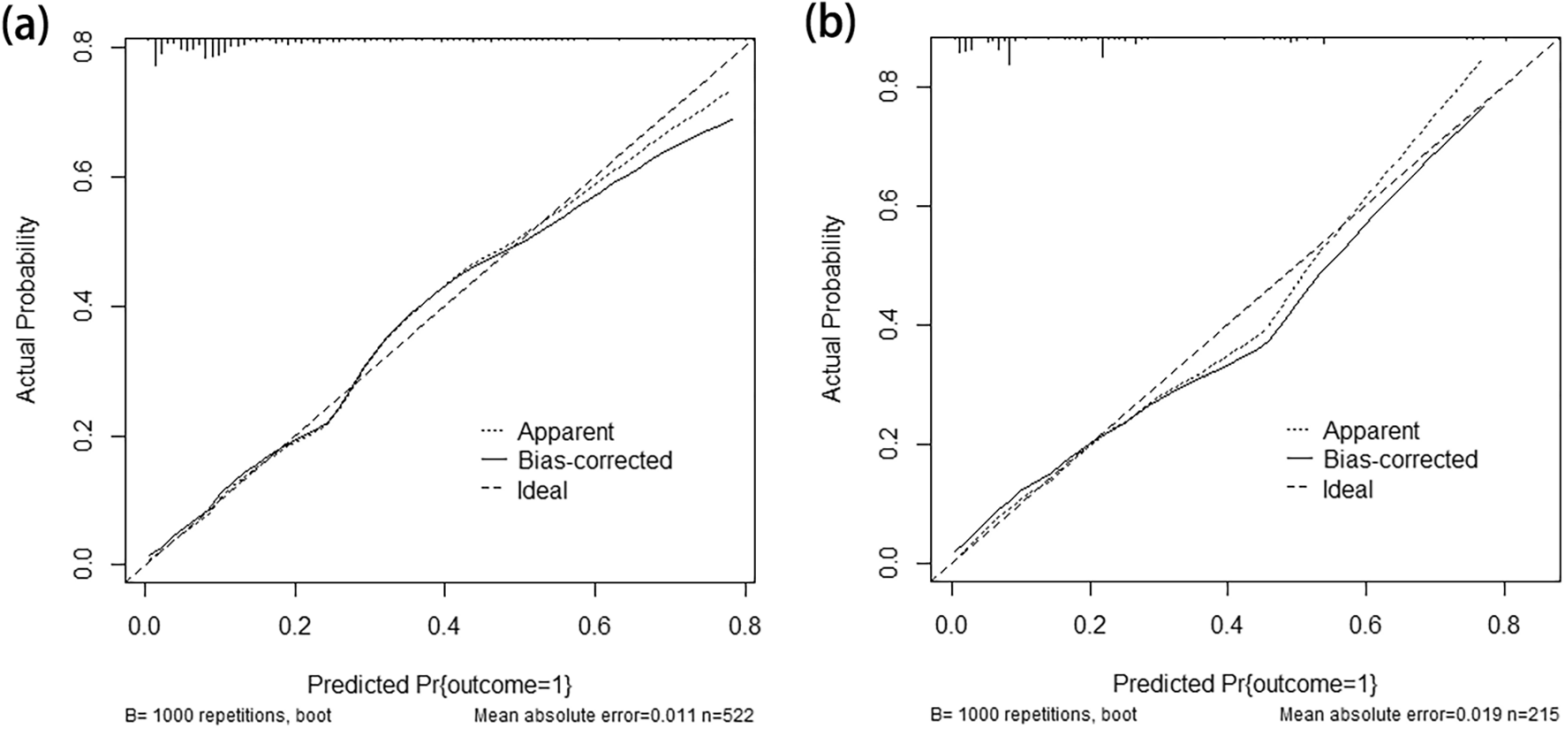
Nomogram calibration curves in the primary (**a**) and external validation (**b**) cohorts (bootstrap method using 1000 repetitions)

The AUC for the nomogram in the primary cohort was 0.817 (95% CI 0.767–0.866) (Fig. 3a), indicating that the nomogram effectively predicted LNM risk in BUC patients. According to the Hosmer–Lemeshow goodness-of-fit test, the nomogram had a P = 0.285, indicating a good logistic regression model fit. The corrected C-index was 0.805 after 1000 bootstrap resampling, demonstrating little change from the primary C-index value (0.817). The calibration curve (Fig. 4a) closely resembled the standard curve, demonstrating that the nomogram had a good level of reproducibility and reliability.

### External validation of the nomogram

After applying the established internal nomogram model to the validation cohort, the AUC was 0.825 (95% CI 0.752–0.897) (Fig. 3b), demonstrating that the nomogram exhibited effective predictive accuracy in the external validation cohort. According to the Hosmer–Lemeshow goodness-of-fit test, the nomogram had a P = 0.480 in the validation cohort, indicating a good logistic regression model fit. The corrected C-index was 0.799 after 1000 bootstrap resampling, indicating little change from the C-index value (0.825) of the external validation cohort. In addition, the calibration curve (Fig. 4b) closely resembled the standard curve, demonstrating that the nomogram also performed well in the external validation cohort.

### Clinical application value of the nomogram

**Fig. 5:**
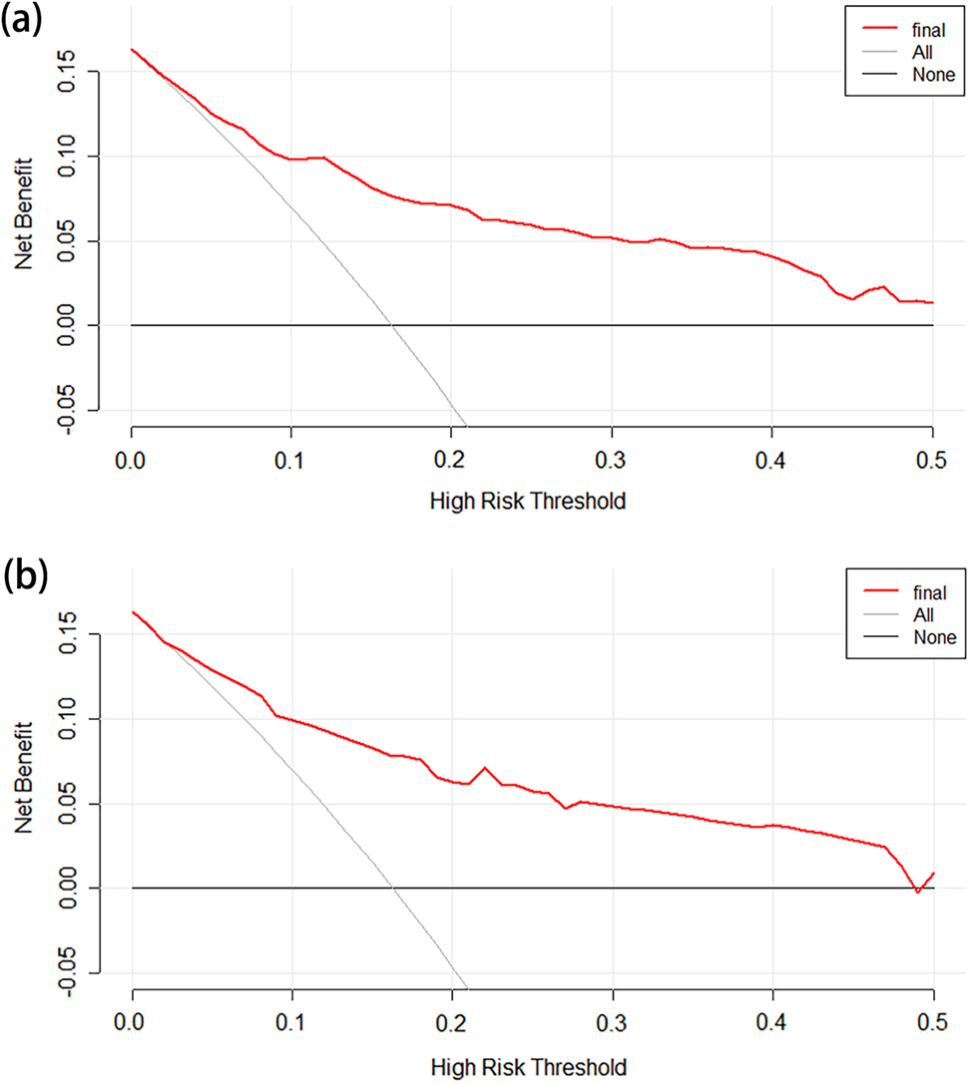
Nomogram decision curves in the primary (**a**) and external validation (**b**) cohorts; the x-axis indicates the threshold probability, while the y-axis indicates the net benefit. The grey line indicates all patients with LNM and the blue line indicates all patients without LNM. *LNM* lymph node metastasis

**Fig. 6:**
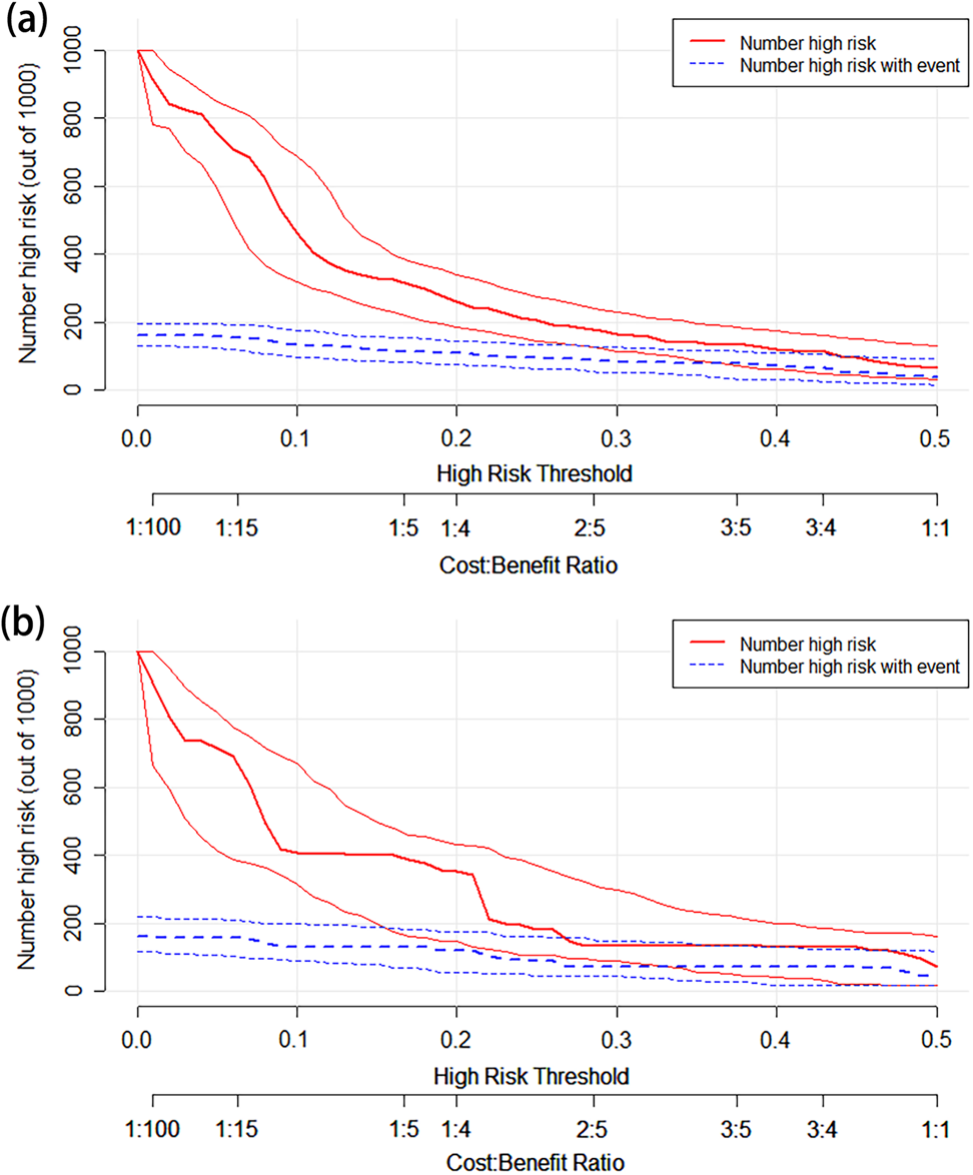
Nomogram clinical impact curves in the primary (**a**) and external validation (**b**) cohorts. The red curve indicates the number of patients who were classified as having LNM at each threshold probability according to the nomogram, while the blue curve indicates the number of patients with true LNM at each threshold probability

Results of the DCA for predicting LNM in BUC, showed that the model offered a clinical benefit in the primary cohort at a threshold of between 0.05 and 0.50 (Fig. 5a). Moreover, DCA demonstrated that the nomogram was helpful in predicting LNM in BUC patients when the threshold probability was 0.05–0.45 in the external validation cohort (Fig. 5b). The clinical impact curve showed that the nomogram had a good clinical application value in differentiating BUC patients with LNM and those without in both the primary (Fig. 6a) and validation (Fig. 6b) cohorts.

## Discussion

LNs are the most common site of bladder cancer metastasis. However, patients with pathological N1–3 tumor were reported to have a significantly shorter 5-year overall survival than those with pN0, indicating that LNM in BUC is associated with a poor prognosis (Karl et al. 2009; Zehnder et al. 2014). Because numerous studies have recognized LNM as the most valuable independent prognostic marker for survival outcomes in BUC, the sensitive and accurate identification of LNM in BUC is of crucial importance (Kawada and Taketo 2011). Results from previous LNM mapping studies have revealed the presence of malignant LNs outside the region of standard LND in a group of patients with LNM (Dorin et al. 2011; Wang et al. 2014). Because extended LND is more challenging to perform and is associated with more perioperative complications than standard LND, it should be used only when strictly necessary in clinical practice. The preoperative prediction of LNM would enable clinicians to choose between extended or standard LND and bladder-sparing treatments or RC, for the benefit of BUC patients.

Computed tomography (CT) and magnetic resonance imaging (MRI) are the imaging methods most commonly used to evaluate whether abdominal and pelvic LNs contain evidence of tumor involvement. However, both CT and MRI have difficulty in identifying normal-sized or minimally-enlarged metastatic modes, thus, limiting the accuracy of LNM identification (Witjes et al. 2021). Several studies have demonstrated that conventional cross-sectional imaging techniques, including CT and MRI, have high specificity but low sensitivity when it comes to identifying LNM. For example, Brunocilla et al. reported that the specificity of enhanced-CT in identifying LNM in BUC was 89%, but the sensitivity was only 14% (Brunocilla et al. 2014). One recent study also found that there was only 64.9% agreement between the N stage identified using conventional cross-sectional imaging and the pathologic N stage; again, high specificity (84%) and low sensitivity (30%) was observed (Lonati et al. 2022). Data from our primary (specificity = 90.6%; sensitivity = 36.5%) and external validation (specificity = 95.0%; sensitivity = 25.7%) cohorts echoed these findings. Although the most advanced imaging techniques, such as positron emission tomography (PET)-CT, had a higher sensitivity and a similar specificity to CT and MRI, they still could not accurately predict LNM in BUC patients (Brunocilla et al. 2014; Einerhand et al. 2020; Ha et al. 2018). Besides, the high cost of PET-CT has limited its widespread use in the preoperative examination of BUC patients. Thus, the aim of the present study was to develop an accurate nomogram to predict LNM in BUC, by including common preoperative imaging parameters and other readily accessible clinical data. Our univariate analyses revealed that BUC patients with pathologically confirmed LNM had significantly more possibility of hydronephrosis, extravesical invasion, LNM on imaging, and enlarged tumors. The multivariate logistic regression analysis then showed that extravesical invasion, LNM on imaging, and tumor size were independent risk factors for LNM in BUC. A study using data from the SEER database also identified tumor size as an independent predictor of LNM in BC (Tian et al. 2021). Moreover, LNM (detected using imaging) was incorporated into two other nomograms, which were based on radiomics signatures, as a significant LNM risk factor (Wu et al. 2017, 2018a, b). Although T stage was reported as independent predictor of LNM in BC by several groups (Shariat et al. 2012; Tian et al. 2021; Schuettfort et al. 2022), the staging accuracy of CT, as the most common radiologic imaging tool in BC, was low. Therefore, in the present study, we selected the “presence or absence of extravesical invasion” parameter instead of “T stage”, and eventually identified it as an independent predictor of LNM in BUC.

To obtain an accurate histopathological diagnosis and reliable staging data, TURBT is typically recommended before RC; moreover, the resection specimen should contain bladder muscle tissue (Witjes et al. 2021). The univariate analysis showed that BUC patients with LNM had significantly higher grade tumors and more infiltration, with little papillary tumor presence, compared with LNM-negative BUC patients. Meanwhile, the multivariate logistic regression analysis identified tumor grade and infiltration as the most significant pathological risk factors for LNM in BUC. Tian et al. and Kim et al. also used TURBT pathological data to identify tumor grade as a predictor of LNM in BC (Tian et al. 2021; Kim et al. 2015). Although the risk of LNM was reported to be significantly higher in MIBC patients than in NMIBC patients (Karakiewicz et al. 2006; Tian et al. 2021), we discovered no statistically significant difference in the extent of muscle invasion between these groups in our study. This might be because the staging accuracy of TURBT pathological examination is low. This is evidenced by the fact that 25–51% patients who were diagnosed with NMIBC following TURBT were upstaged to MIBC at RC (Shariat et al. 2007; Svatek et al. 2011; Turker et al. 2012). Thus, the reason for identifying infiltration as an independent risk factor for LNM in BUC might be because it is accurately reported following TURBT. As for urothelial variants, one recent published study indicated that non-muscle invasive BUC patients with variant histology had significantly worse disease-free survival and cancer-specific survival (Lopez-Beltran et al. 2022). We explored the relationship between urothelial variants and LNM, and we discovered no statistically significant difference between patients with and without urothelial variants. One article demonstrated that BUC patients with urothelial variants had better or worse prognosis compared with pure BUC depending on different specific variants (Claps et al. 2023). Therefore, the influence of each variant in LNM should be further explored in the future. In the present study, we chose to include the papillary status of BUC tumors as a parameter because tumors with a papillary component are associated with slower BC progression (Beijert et al. 2023). Our univariate analysis of both BUC cohorts showed that tumors with a papillary component were significantly associated with reduced possibility of LNM; however, it was not an independent predictor in the final model. Because immunohistochemistry is not a mandatory tool in BC diagnosis (Comperat et al. 2021), the presence of lymphovascular invasion (LVI) was not accurately reported for many of the TURBT specimens included in our study. Thus, we did not include incomplete LVI variable, even if it was reported to be associated with a worse BUC prognosis and increased LNM risk (Mari et al. 2019; Martin-Doyle et al. 2015).

The diagnostic and prognostic value of preoperative inflammatory biomarkers and other laboratory measurements in BUC has been previously reported. For instance, Tang et al. found that preoperative NLR, SII, and derived NLR were significantly different between BUC patients with LNM and those without (Tang et al. 2020). D'Andrea et al. also demonstrated that MLR and NLR were associated with LNM in BUC (D'Andrea et al. 2017). Other measurements, such as hemoglobin and PLR, are also linked to poor prognosis or LN status in BUC (Pang et al. 2016; Sejima et al. 2014; Viers et al. 2014). In the present study, we used preoperative laboratory measurements and inflammatory biomarkers, including NLR, PLR, MLR, NPR, and SII, to predict LNM in BUC. Two previously published studies identified MLR, fibrinogen, and NLR as preoperative predictors of LNM in BUC and included them in nomograms (Ou et al. 2020; Schuettfort et al. 2022). Although most laboratory measurements (including MLR, fibrinogen, and NLR) included in our study were significantly different between BUC patients with LNM and those without in the univariate analysis, serum creatine was the only variable capable of independently predicting LNM in BUC before RC in the multivariate analysis. Besides, a recent multicenter study indicated that low albumin-to-fibrinogen ratio was also associated with LNM at time of RC (Claps et al. 2021). Although both albumin and fibrinogen were significant in our univariate analysis, they were not significantly different between patients with and without LNM in multivariate logistic regression analysis. The opposite conclusion indicated the controversy of these markers, which should be further analyzed in research with larger sample size.

Thus, the final nomogram to preoperatively predict LNM before RC in patients with BUC was based on tumor grade, infiltration, extravesical invasion, LNM on imaging, tumor size, and serum creatinine levels. To ensure the clinical relevance of our nomogram, we included variables which were representative and readily available from the pathological analysis of TURBT specimens, imaging data, and laboratory measurements. The nomogram had an AUC of 0.817 (95% CI 0.767–0.866) in the primary cohort and an AUC of 0.825 (95% CI 0.752–0.897) in the external validation cohort, suggesting that it exhibited a good level of predictive accuracy. The calibration curves of the primary and validation cohorts indicated that the model performed consistently well during internal validation and external validation. Moreover, the DCA results and clinical impact curves of both cohorts showed that the nomogram was highly clinically applicable.

Our study had some limitations. First, the retrospective study design can lead to inaccurate data selection and the introduction of other potential confounders. Second, although the internal and external cohort validation results were consistent and stable, the sample size was small, especially for the external validation cohort. Third, because we were limited by the quality of the pathological data derived from TURBT samples, we did not collect LVI or carcinoma in situ data for inclusion in the nomogram. The limited sample size also restricted us to analyze the influence of each variant of urothelial variants in LNM in BUC patients. Finally, some new model constructing algorithms, such as machine learning, have been used to predict the risk of LNM in prostate cancer and renal cell carcinoma (Li et al. 2022; Sabbagh et al. 2023; Zhang et al. 2023). Our model was developed using traditional univariate and multivariate logistic regression analyses. Further research is needed to determine the comparative efficacy of conventional and modern models in preoperatively predicting LNM in BUC.

## Conclusion

Here, we used pathologic information from TURBT specimens, imaging data, and laboratory measurements to develop a nomogram, which comprised the tumor grade, infiltration, extravesical invasion, LNM on imaging, tumor size, and serum creatinine parameters to preoperatively predict LNM in BUC. This nomogram displayed high levels of accuracy, reliability, and clinical applicability following internal and external validation.

## Data Availability

The datasets used and analyzed during the current study are available from the corresponding author upon reasonable request.
